# Complete Genome Sequence of a USA100 Methicillin-Resistant Staphylococcus aureus Strain

**DOI:** 10.1128/MRA.00006-19

**Published:** 2019-03-14

**Authors:** George E. Chlipala, Zhengdeng Arden Lei, Mark Maienschein-Cline, Kevin Kunstman, Koh Okamoto, Stefan J. Green, Kyle J. Popovich

**Affiliations:** a Research Informatics Core, Research Resources Center, University of Illinois at Chicago, Chicago, Illinois, USA; b Sequencing Core, Research Resources Center, University of Illinois at Chicago, Chicago, Illinois, USA; c Division of Infectious Disease, Department of Medicine, Rush University Medical Center, Chicago, Illinois, USA; University of Maryland School of Medicine

## Abstract

We report the genome sequence of a methicillin-resistant Staphylococcus aureus (MRSA) strain, isolated from a surgical intensive care unit. This completely closed genome of a USA100 isolate contains a major chromosome and a plasmid and will serve as a reference genome for genetic analysis of MRSA strains.

## ANNOUNCEMENT

Although MRSA strains were originally seen solely in health care settings, over the past 15 to 20 years, strains have been identified in the community among otherwise healthy individuals. Using pulsed-field gel electrophoresis, researchers identified USA300 as the predominant community strain and USA100 as the strain traditionally found in hospital settings (1) and often isolated from patients with prior health care exposures or comorbidities. As part of research examining MRSA in health care settings, an isolate (strain 30-47) was recovered from a surveillance swab from the anterior nares of a patient in the surgical intensive care unit at Rush University Medical Center in Chicago, IL, in 2015. The swabbing was performed as part of an infection control research project, and research staff collected the specimen. The patient’s comorbidities included lung disease, including lung cancer. We sought to generate a closed reference assembly from this isolate for comparative purposes in a large analysis of hospital-associated MRSA strains.

A flocked nylon swab (ESwab, Becton-Dickenson [BD], New Jersey) premoistened with Amies medium was used to obtain the organism. The swab was inoculated into 5 ml tryptic soy broth plus 6.5% NaCl, incubated for 48 h, and then plated onto MRSA Spectra agar (Remel, Kansas) and incubated for 24 h. MRSA was confirmed with a catalase test and a slide coagulase test. Genomic DNA from the isolate was recovered using a Maxwell 16 instrument (Promega) and sequenced using a Nextera XT DNA library preparation kit (Illumina) and an Illumina NextSeq 500 instrument (2 × 150 paired-end [PE] reads). Nanopore sequencing libraries were generated using the Oxford Nanopore rapid barcoding sequencing SQK-RBK004 protocol and sequenced on a SpotON flow cell vR9 (catalog number FLO-MIN106) for 48 h. Reads were processed for base-calling using Albacore v2.1.10 (2). DNA extraction, library preparation, and sequencing were performed at the University of Illinois at Chicago Sequencing Core (UICSQC).

Prior to assembly, Nanopore data were filtered to remove reads shorter than 1,000 bases and trimmed to remove adapters using Porechop v0.2.3 (3). These reads were assembled using Canu v1.5 (4) with the following parameters: genomeSize, 5 m; corMhapSensitivity, high; corMinCoverage, 2; and errorRate, 0.035. This yielded two contigs with a total sequence length of 2,804,978 bases. Initial error correction of the assembly was performed using Minimap2 v2.6 (5) and Racon (git commit 0834442ab980b10f7b0805570d1edab19d268767) (6) with the Nanopore data. The error-corrected assembly was tested for possible circularity using Circlator v1.5.5 (7), executing “all” steps with the following parameters: merge_min_id, 85; merge_breaklen, 1000; assembler, canu; and bwa_opts, -x ont2d. The circularized contigs were then polished in an iterative fashion using Illumina data. Briefly, the reads were aligned to the contigs using the Burrows-Wheeler Aligner MEM algorithm (BWA MEM) v0.7.15 (8), and the mpileup output from the alignment was processed by custom script to generate a corrected or polished assembly. The polished assembly was used for further alignment and polishing processes that were repeated until no changes could be detected (10 rounds).

The draft genome sequence of MRSA USA100 strain 30-47 consists of a single closed chromosome (2.76 Mbp, 32.86% G+C content) and one plasmid (27,086 bp, 27.22% G+C content). A total of 2,670 coding sequences, 19 rRNA features, 59 tRNAs, and 4 noncoding RNAs (ncRNAs) were predicted and annotated using the NCBI Prokaryotic Genome Annotation Pipeline (9). Genome alignments of strain 30-47 using MUMmer v3.23 (10) revealed 99.92% sequence identity with 98.87% coverage with the closed genome of USA100 S. aureus strain N315 (accession number NC_002745) (11) and 99.95% sequence identity with 98.02% coverage with the draft genome of USA100 S. aureus strain 209 (accession number NTCY00000000) (12).

### Data availability.

This project has been deposited in DDBJ/EMBL/GenBank under the accession numbers CP029474 and CP029475. The version described in this paper is the first version. Raw sequence data have been deposited in the NCBI Sequence Read Archive under accession numbers SRR7179412 and SRR7179413 for the Illumina and Nanopore data, respectively.
